# Comprehensive Phenotypic Characterization and Genomic Analysis Unveil the Probiotic Potential of *Bacillus velezensis* K12

**DOI:** 10.3390/ani15060798

**Published:** 2025-03-11

**Authors:** Yingying Tang, Tian Li, Yihong Huang, Liangliang Wu, Xiaobo Liu, Ruichao Yue, Jianmin Yuan

**Affiliations:** 1 State Key Laboratory of Animal Nutrition and Feeding, College of Animal Science and Technology, China Agricultural University, Beijing 100193, China; sy20243041032@cau.edu.cn (Y.T.); huangyh@cau.edu.cn (Y.H.); 2 College of Animal Science and Technology & College of Veterinary Medicine, Zhejiang A&F University, Hangzhou 311300, China; hsjimmyf@hotmail.com; 3 Ningxia Eppen Biotech Co., Ltd., Yinchuan 750100, China; wuliangliang@eppen.com.cn (L.W.); liuxiaobo1@eppen.com.cn (X.L.); 4 Department of Basic Veterinary Medicine, College of Veterinary Medicine, China Agricultural University, Beijing 100193, China

**Keywords:** *Bacillus velezensis*, probiotic, antimicrobial activity, genome analysis

## Abstract

*Bacillus* spp. have emerged as pivotal sources of probiotic preparations, garnering considerable attention in recent years owing to their vigorous bacteriostatic activity and antimicrobial resistance. This study aimed to investigate these probiotic characteristics in depth and verify the safety of *Bacillus velezensis* K12, a strain isolated from broiler intestine. The K12 strain was identified as *Bacillus velezensis* based on its morphology and 16S rDNA sequence homology analysis. Subsequently, *B. velezensis* K12 was evaluated for acid resistance, bile salt resistance, gastrointestinal tolerance, drug sensitivity, and antimicrobial activity. Additionally, whole-genome sequencing technology was employed to dissect its genomic components further, aiming to explore its potential applications as a probiotic strain. *B. velezensis* K12 was sensitive to six antibiotics and had acid tolerance. Furthermore, it showed potent antimicrobial activity against a wide range of pathogenic bacteria, including *Escherichia coli* (*E. coli*), *Staphylococcus aureus*, *Salmonella*, *Clostridium perfringens*, *Bacillus cereus*, and *Vibrio parahaemolyticus*. The complete genome sequencing of *B. velezensis* K12 revealed a genomic length of 3,973,105 base pairs containing 4123 coding genes, among which 3973 genes were functionally annotated. The genomic analysis identified genes associated with acid and bile tolerance, adhesion, antioxidants, and secondary metabolite production, whereas no functional genes related to enterotoxins or transferable antibiotic resistance were detected, thereby confirming the probiotic properties of *B. velezensis* K12. *B. velezensis* K12 exhibits broad-spectrum bacteriostatic activity and in vitro safety, positioning it as a potential candidate strain for developing probiotic *Bacillus* preparations.

## Simple Summary

The increasing utilization of antibiotics has sparked widespread public concern, and the search for antibiotic alternatives has become a research hotspot. *Bacillus* spp. have emerged as pivotal sources of probiotic preparations, garnering considerable attention in recent years owing to their strong bacteriostatic activity and antimicrobial resistance. In this study, a strain of *Bacillus velezensis* K12 was screened from broiler intestine and assumed to be probiotic. The results of an in vitro assay and whole-genome sequencing showed that K12 possesses potent bacteriostatic properties and exhibits safety in vitro, positioning it as a promising candidate for further probiotic development.

## 1. Introduction

The increasing utilization of antibiotics has sparked widespread public concern since 1990. Several alternatives to antibiotics have been proposed to control the problems associated with their overuse, such as bacteriocins [1], oligosaccharides [2], enzymes [3], and probiotics [4]. The International Scientific Association for Probiotics and Prebiotics (ISAPP) define probiotics as “live microorganisms that, when administered in adequate amounts, confer a health benefit on the host” [5]. The main bacterial probiotics commonly used are *Lactobacillus*, *Bifidobacterium*, and *Bacillus* [6]. *Bacillus* is a Gram-positive bacterium that produces spores. Compared to *Lactobacillus* and *Bifidobacterium*, *Bacillus* has higher acid resistance and better stability during heat treatment and cold storage [7]. *Bacillus* species can secrete numerous metabolites and peptides with antimicrobial activity, which exert various effects, including antibacterial, antifungal, and growth-promoting impact [8].

*Bacillus velezensis* was initially isolated from the Vélez River in Torredelmar, Málaga, southern Spain [9]. It can be readily isolated and cultured and is widely found in nature [10]. The *Bacillus velezensis* strain exhibits a notable capacity to produce secondary metabolites with potent antibacterial characteristics, including antibiotic lipopeptides, polyketides, and peptides [11]. Given its extensive natural distribution and the enrichment of its metabolites, *Bacillus velezensis* has gained increasing recognition in recent years as a probiotic [12]. *Bacillus velezensis* has been widely studied as a biocontrol agent due to its antagonistic potential against various phytopathogenic fungi [13,14,15]. Moreover, numerous studies have described the probiotic properties of different *Bacillus velezensis* strains in the aquaculture and poultry industries [16,17,18,19]. Several studies have also supported the safety and promise of *Bacillus velezensis* strains for application as human oral probiotics [20,21].

Whole-genome sequencing provides tremendous insight into the potential properties of microbes as probiotics, as well as valuable information regarding their use [22]. The genomes of *Bacillus velezensis* from diverse sources, including potato tubers [23], termite gut [24], and soil [25], have been thoroughly described, thereby elucidating the underlying mechanisms responsible for its probiotic attributes.

In the current study, *B. velezensis* K12 isolated from broiler intestine was investigated for its probiotic potential, focusing on its growth characteristics, resistance to acid and bile salts, antibiotic susceptibility, and in vitro bacteriostatic properties. We also performed a complete genome analysis of *B. velezensis* K12 to explore its genetic determinants concerning its probiotic properties. This study provides valuable insights into the genome and phenotypic features of *B. velezensis* K12, thereby identifying it as a potent antimicrobial agent in future research and animal husbandry.

## 2. Materials and Methods

### 2.1. Isolation, Purification, and Characterization of Strains

Strain K12 was isolated from broiler intestine. The indicator strains for the in vitro studies were *E. coli* K88, *E. coli* CVCC25922, *Staphylococcus aureus* CVCC1822, *Salmonella* CVCC519, *Clostridium perfringens* CVCC66, *Bacillus cereus* CICC21290, and *Vibrio parahaemolyticus* CICC23924, which were all provided by the Animal Nutrition Process Development Department of the R&D Center of Ningxia Yipin Biotechnology Co., Ltd. (Yinchuan, China).

Strain K12 was inoculated onto Luria–Bertani (LB) solid medium using the line dilution method and incubated inverted at 37 °C for 14 h to observe the morphology of the colonies. Single colonies on LB medium were picked for Gram staining, and the morphological characteristics of the bacteria were examined under an upright microscope (Olympus CX23, Yijingtong Optics Technology Co., Shanghai, China). Subsequently, single colonies on LB medium were picked and inoculated into LB liquid medium (Beijing YiKeRan Biotechnology Co., Beijing, China). They were then incubated in a constant temperature shaker (Shanghai Jinghong Laboratory Equipment Co., Shanghai, China) at 37 °C and 220 rpm for 14 h. Centrifugation was performed to collect the bacterial bodies, which were then forwarded to Shanghai Parsonage Bio-technology Co. (Shanghai, China). for 16S rDNA gene sequencing. PCR amplification was conducted with 27F: 5′-AGAGTTTGATCCTGGCTCAG-3′ and 1492R: 5′-GGTTACCTTGTTACGACTT-3′ primers [26]. The PCR reaction mixture (50 μL) included 5 μL of 10× Buffer, 1 μL of Taq polymerase, 1 μL of dNTPs, 1.5 μL of each primer (upstream and downstream), and 1 μL of template. The PCR reaction program was as follows: pre-denaturation at 95 °C for 5 min, denaturation at 95 °C for 30 s, annealing at 58 °C for 30 s, extension at 72 °C for 1 min and 30 s, repeated for 35 cycles, followed by a final extension at 72 °C for 7 min, and storage at 4 °C. The amplified products were analyzed via 1.0% agarose gel (Beijing YiKeRan Biotechnology Co., Beijing, China) electrophoresis and sequenced. The obtained sequences were submitted to the NCBI database for BLAST comparison (BLAST v2.13.0) [27]. Sequences from 11 closely related species were retrieved, and a 16S evolutionary tree was constructed using MEGA11, using the neighbor-joining method, with a bootstrap value of 1000 [28], based on multiple sequence alignments performed with MAFFT software [29].

### 2.2. Determination of Strain Biology

#### 2.2.1. Determination of the Growth Curve of Strain K12 Versus the pH Change Curve

Referring to Renschler et al. [30] with minor modifications, the K12 strain was activated and cultivated in LB liquid medium for 14 h. The cultured bacterial suspension was transferred to fresh LB liquid medium at a 1% inoculum ratio and incubated in a shaker at 37 °C and 220 r/min for 36 h. Samples were collected every 2 h, and the absorbance at 600 nm and the pH of the bacterial culture were measured.

#### 2.2.2. Hemolysis Analysis

The activated K12 strain was cultured on sheep blood agar plates (Beijing YiKeRan Biotechnology Co., Beijing, China), and the results were observed after 14 h of incubation at 37 °C [31]. The formation of a green circle around the colonies indicated α-hemolytic activity, while the formation of a transparent circle indicated β-hemolytic activity. In contrast, the absence of a transparent circle indicated γ-hemolytic activity.

#### 2.2.3. Acid Resistance Analysis

To study the acid resistance of the strains, the method of Soni et al. [31] was referred to and slightly modified. The pH of the LB liquid medium was adjusted to 1.5, 2.5, 3.5, 4.5, 5.5, and 6.5 using 1 mol/L HCl. The activated bacterial broth was inoculated into LB liquid medium with different pH values at an inoculum rate of 1%, with pH 7.1 inoculated and non-inoculated LB liquid medium as the control. The samples were incubated at 37 °C and 220 r/min for 24 h. After incubation, samples were taken at 0, 2, 4, 8, 12, and 24 h, and the absorbance was measured at a wavelength of 600 nm using a UV spectrophotometer (TU-1950, Beijing Puxi General Instrument Co., Beijing, China).

#### 2.2.4. Bile Resistance Analysis

The bile resistance of the strains was determined by the method of Reyes-Cortes et al. [32] with slight modifications. The corresponding masses of bile salts were weighed and dissolved into sterile LB liquid medium, and 0.00%, 0.02%, 0.04%, 0.06%, 0.08%, 0.10%, 0.20%, and 0.30% bile salts were prepared. The bile salts were filtered to remove bacteria. The LB liquid medium without bacteria was used as a control. The activated bacterial solution was added to the LB liquid medium with varying bile salt concentrations at an inoculum rate of 1% and then incubated at 37 °C and 220 r/min for 24 h. After incubation, samples were taken at 0, 2, 4, 8, 12, and 24 h, and the absorbance was measured at a wavelength of 600 nm using a UV spectrophotometer.

#### 2.2.5. Determination of Tolerance to Simulated Gastrointestinal Fluids

To determine the tolerance of strain K12 to simulated gastrointestinal fluids, the method of Tanvi et al. [33] was used with minor modifications. The activated bacterial solution was centrifuged at 3000 r/min for 10 min, and the bacterial bodies were collected and washed twice with sterile PBS buffer (pH 7.3–7.5) to remove the residual medium. The bacteria were resuspended in 5.0 mL of PBS, and 1.0 mL of each bacterial suspension was separately mixed with 9.0 mL of sterile simulated artificial gastric fluid (containing 1% pepsin, pH 2.0) and 9.0 mL of sterile simulated artificial intestinal fluid (containing 0.2% trypsin and 1.2% bovine bile salts, pH 8.0). The simulated gastrointestinal fluids were purchased from Beijing Yikeran Biotechnology Co (Beijing, China). The mixtures were cultured at 37 °C. After 3 h of both initial and co-culturing, the viable bacteria were counted on agar plates, and the relative content was calculated.

#### 2.2.6. Antibiotic Susceptibility Testing

The disk diffusion method was used for the drug sensitivity test, with results interpreted according to the American Society for Clinical Laboratory Standardization (CLSI) 2021 standards [34]. A fresh bacterial suspension with a concentration of 1.0 × 10^8^ CFU/mL (100 μL) was inoculated onto an LB agar plate. Drug-sensitive tablets containing cefotaxime, florfenicol, ciprofloxacin, cotrimoxazole, doxycycline, and gentamicin were placed onto plates coated with the bacterial solution. The tablets were gently pressed to ensure they adhered tightly to the plate, and then inverted and placed on the LB plate, and the inhibition zone diameters were measured after incubation at 37 °C for 24 h.

#### 2.2.7. Determination of Bacteriostatic Capacity

The bacteriostatic capacity of strain K12 was determined using a modified version of the Oxford cup double plate method [35]. *E. coli* K88, *E. coli* CVCC25922, *Staphylococcus aureus* CVCC1822, *Salmonella* CVCC519, *Bacillus cereus* CICC21290, *Clostridium perfringens* CVCC66, and *Vibrio parahaemolyticus* CICC23924 were used as the indicator bacteria, which were inoculated into LB liquid medium, RCM liquid medium, and high-salt LB liquid medium containing 3.5% NaCl, respectively, to make suspensions containing 1 × 10^8^ CFU/mL indicator bacteria. Strain K12 was inoculated into LB liquid medium and incubated at 37 °C and 220 r/min for 14 h, and then diluted 10, 100, 1000, 4000, 8000, and 10,000 times via gradient dilution with sterile water. Pure agar plates (containing 1.8% agar) were prepared by placing 8 mm diameter Oxford cups (Beijing YiKeRan Biotechnology Co., Beijing, China) evenly on the solidified agar plates. To LB solid medium/anaerobic broth solid medium/high-salt LB solid medium (containing 1.8% agar) at around 50 °C, add a 1% (*v*/*v*) suspension of indicator bacteria; mix the two and pour onto an agar plate (containing 1.8% agar). After the medium solidified, the Oxford cup was pulled out using sterile forceps and 200 μL of the above-treated strain K12 dilution was injected into each well. After static diffusion for 2 h, the sample was positioned in a 37 °C constant-temperature incubator for 12–20 h. The presence of an inhibition zone was observed, and the size of the inhibition zone was measured using a vernier caliper. Each experimental group was repeated three times.

Strain K12 was cultured in LB liquid medium at 37 °C with agitation at 220 r/min for 14 h. The culture was then centrifuged at 3000 rpm for 10 min to separate the supernatant from the sediment. After that, the supernatant was filtered through a 0.22 μm microfiltration membrane to remove bacteria, and the sediment was washed with sterile PBS buffer (Beijing YiKeRan Biotechnology Co., Beijing, China) 3 times to remove residual medium and supernatant and then resuspended in PBS. The indicator bacteria were subjected to the same as the above, and the inhibition effect of strain K12 whole culture, supernatant, and sediment was determined by the Oxford cup double plate method. Each set of experiments was repeated three times.

Strain K12 was cultured in LB liquid medium, incubated at 37 °C and 220 r/min on a shaker (SPH-110X24, Shanghai Shiping Experimental Equipment Co., Shanghai, China) for 14 h, and then boiled to inactivate it. The bacteriostatic ability of strain K12 after inactivation was assessed using the Oxford cup double plate method, with *E. coli* K88 serving as an indicator bacterium. Each group of experiments was repeated three times.

#### 2.2.8. Whole-Genome Sequencing and Annotation

The genomic DNA of strain K12 was extracted utilizing the TruSeq^TM^ DNA Sample Prep Kit (Tiangen, Beijing, China). Then, 1% agarose gel electrophoresis and a NanoDrop 2000 spectrophotometer (Thermo Fisher Scientific, Waltham, MA, USA) were used to assess the quality and concentration of the extracted DNA [36]. The complete genome sequencing of strain *B. velezensis* K12 was performed in two stages: second-generation sequencing using the Illumina NovaSeq 6000 platform (Illumina, San Diego, CA, USA) and third-generation sequencing using the Oxford Nanopore platform (Oxford Nanopore, Oxford, UK) [37]. To generate second-generation data, libraries were prepared from the samples and sequenced. Raw data quality was assessed and controlled using fastp software (v0.23.1), including splice contamination removal, length filtering, reads quality filtering, and fuzzy base N filtering [38]. After data filtering, the number of high-quality reads of strain K12 was 7,011,760, and the percentage of high-quality reads in the downstream reads was 99.53%. The number of high-quality reads was 1,058,327,828 bp, and the percentage of high-quality reads in the downstream bases was 99.49%. SOAPec [39] was used to quality-correct all reads according to the K-mer frequency, and the K-mer used for quality correction was 19. Third-generation sequencing data were assembled using Unicycler [40], followed by correction of the assembly results using Pilon software (v1.24) based on high-quality second-generation data [41]. Finally, the spliced complete sequence was obtained.

Average Nucleotide Identity (ANI) is an important parameter based on the whole-genome sequences of species, and is used to determine the genetic relatedness between species by analyzing and comparing homologous gene sequences [42]. It provides an intuitive representation of the phylogenetic distance between species. Using the complete sequences from the assembly results, fastANI software (v1.33) (https://github.com/ParBLiSS/FastANI/releases, accessed on 19 September 2024) was employed to search the Up-to-Date Bacterial Core Gene (UBCG) database for 20 closely related species [43]. ANI analysis was performed to obtain ANI values, and UBCG software (v1) (https://help.ezbiocloud.net/ubcg-users-manual/, accessed on 19 September 2024) was used to construct a phylogenetic tree based on the core genes of the sample.

The prediction of genome components included identifying protein-coding genes, non-coding RNAs, repetitive sequences, prophages, genomic islands, and clustered regularly interspaced short palindromic repeat (CRISPR) sequences.

Protein-coding genes in the bacterial genome were predicted using GeneMarkS software (v4.32) [44]. tRNA genes were found utilizing tRNAscan-SE [45], while rRNA genes were identified employing Barrnap (https://github.com/tseemann/barrnap, accessed on 17 September 2024). Additional non-coding RNAs were primarily predicted through comparison with the Rfam database (v12.2) [46]. RepeatMasker software (v4.0.7) was employed to predict scattered repeat sequences [47], and TRF software was used for predicting tandem repeat sequences [48]. CRISPR sequences were identified across the genome using CRISPRCasFinder (v4.2.20) [49]. Prophage presence was predicted with PhiSpy (v4.2.21) [50], and gene islands were identified using IslandViewer 4 (v0.2) [51].

To predict gene functions, four distinct databases were utilized: Kyoto Encyclopedia of Genes and Genomes (KEGG) (v111.1) [52], Clusters of Orthologous Groups (COGs) v2.1.12 [53], Gene Ontology (GO) (v2024.9.8) [54], and the Carbohydrate-Active Enzymes database (CAZy) (v12) [55]. The whole genome of strain K12 was searched by BLAST against the above functional databases (https://blast.ncbi.nlm.nih.gov/Blast.cgi, accessed on 19 September 2024). Meanwhile, the predicted genomic sequences were aligned with the Comprehensive Antibiotic Research Database (CARD) (v2023.12) to analyze resistance genes and with the Virulence Factors of Pathogenic Bacteria Database (VFDB) (v2024.03) to annotate virulence genes [56,57]. Additionally, gene clusters for secondary metabolites were identified with the Antibiotics and Secondary Metabolite Analysis Shell (antiSMASH) [58]. Probiotic-related genes were identified through manual extraction from genome annotations and validated via BLASTp analysis against NCBI’s nonredundant protein database (https://blast.ncbi.nlm.nih.gov/Blast.cgi, accessed on 19 September 2024) [59].

### 2.3. Statistical Analysis

The in vitro bacterial inhibitory activity was analyzed with one-way ANOVA, and the in vitro bacterial inhibitory activity data were analyzed with linear regression using SPSS 27.0 software [60]. The results are presented as the mean ± standard deviation, with significance determined at *p* < 0.05. The phylogenetic tree of *B. velezensis* K12 was plotted using MEGA11 software, and the graphs depicting acid and bile salt resistance were prepared using GraphPad Prism 9.5 software.

## 3. Results

### 3.1. Identification of Strain K12

**Figure 1:**
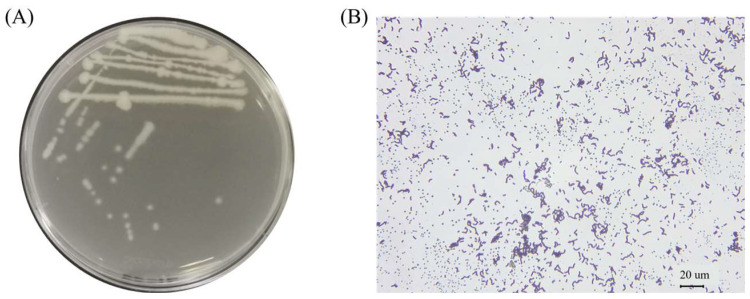
Morphological characteristics of *B. velezensis* K12. (**A**) Colony morphology in LB solid medium. (**B**) Gram-stained bacteria observed under a 400× light microscope.

**Figure 2:**
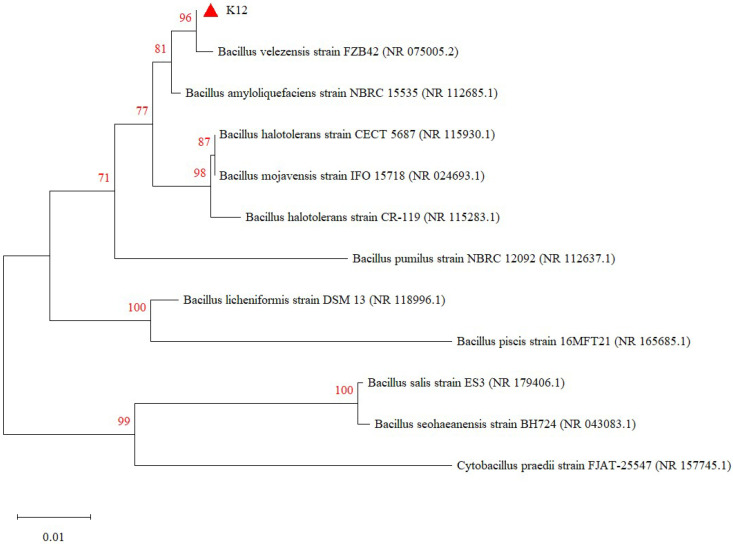
Phylogenetic tree of *B. velezensis* K12 derived from 16S rDNA. The values on the tree reflect the confidence level that the corresponding branch is correct.

**Figure 3:**
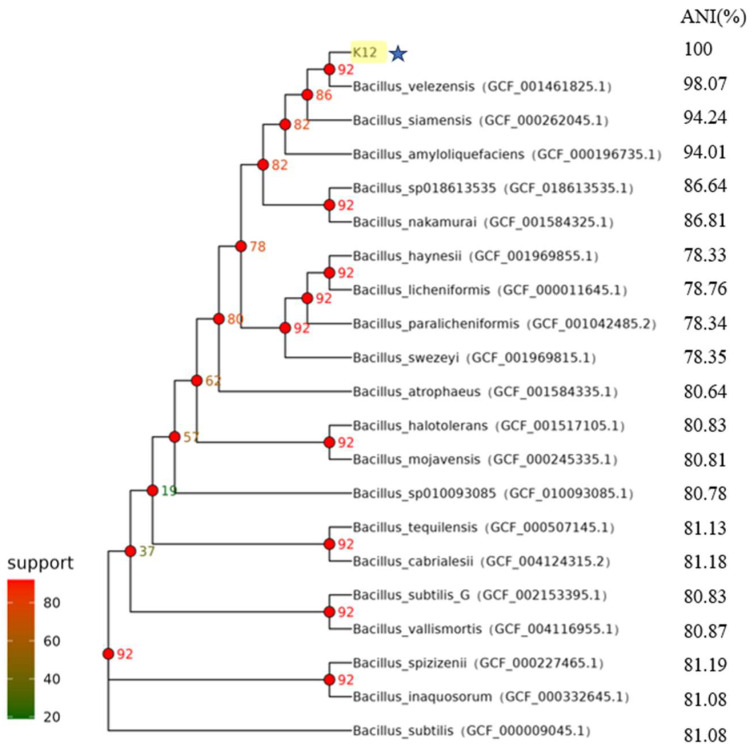
Core genetic evolutionary tree of *B. velezensis* K12 derived from ANI. Values corresponding to the branch node are the number of genes supporting the branch. *B. velezensis* K12 is labelled in yellow and star symbols in the figure.

The test strains were isolated on LB solid medium using the line dilution method, and the inoculated medium was incubated in a constant temperature incubator at 37 °C for 14 h. The morphology of the colonies formed on the medium’s surface was observed. Strain K12 grew well on the LB solid medium, with milky-white colonies and slightly irregular edges (Figure 1A). Strain K12 was Gram-stained and observed under the light microscope as short purple rods, indicating Gram-positive bacteria (Figure 1B). The 16S rDNA sequence was amplified and sequenced, followed by a gene sequence comparison with the NCBI database. Phylogenetic tree analysis indicated that the target strain was the most closely related to *B. velezensis* FZB42 (Figure 2). ANI calculates the percentage of nucleotide identity between two strains by comparing the similarity of their whole-genome sequences. Typically, an ANI value ≥95% indicates that the two strains belong to the same species [42]. The ANI homology indices were calculated between strain K12 and 20 closely related species (Figure 3), and the results showed that strain K12 had the highest homology with the species *Bacillus velezensis* (ANI = 98.07%). Based on the morphological features and molecular analysis, strain K12 was confirmed as *Bacillus velezensis*.

### 3.2. Characterization of the Growth of B. velezensis K12

**Figure 4:**
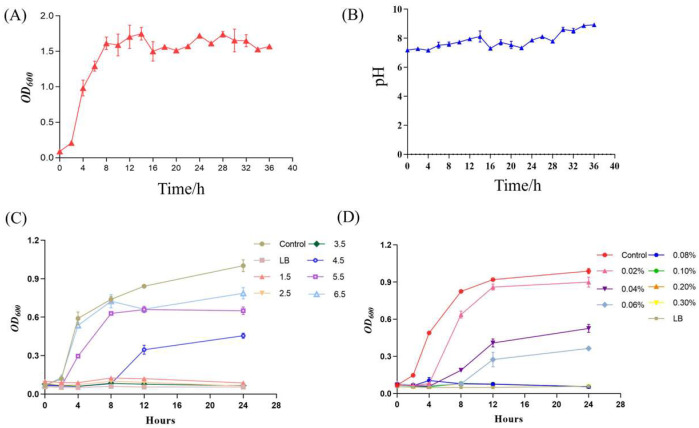
Growth characterization analysis of *B. velezensis* K12. (**A**) Growth curve. (**B**) pH variation curve. (**C**) Acid resistance. (**D**) Bile salt resistance.

*B. velezensis* K12 was inoculated into LB liquid medium to monitor its growth dynamics and pH fluctuations over 36 h. Samples were collected at regular intervals to chart the growth curve (Figure 4A) and the pH variation curve (Figure 4B) of strain *B. velezensis* K12 from the moment of inoculation until 36 h later. During the initial phase, from 0 to 2 h, *B. velezensis* K12 exhibited slow growth. Subsequently, after 2 h, the strain entered the logarithmic growth phase, during which its concentration increased rapidly. This rapid growth continued until 8 h of incubation, when the strain reached the stabilization phase, marking a point where its population no longer increased significantly. Concurrently, the pH value of the medium was measured at various time points, revealing a range of 7 to 8.5 throughout the experiment.

*B. velezensis* K12 exhibited minimal growth within the pH range of 1.5 to 2.5. However, a gradual increase in growth was observed as the pH surpassed 3.5, as illustrated in Figure 4C. Furthermore, as the concentration of bile salts increased, the development of *B. velezensis* K12 was progressively inhibited. Specifically, growth was virtually absent after adding 0.06% bile salts, as shown in Figure 4D. After incubation, no hemolytic ring appeared around a single colony of *B. velezensis* K12 on sheep blood agar, indicating that it was not hemolytic (Supplementary Figure S1). When subjected to an artificial gastric solution for 3 h, *B. velezensis* K12 demonstrated a survival rate of merely 0.01%, indicating almost no growth. Conversely, after being exposed to an artificial intestinal solution for 3 h, the strain exhibited a survival rate of 40.70% (Table 1).

*B. velezensis* K12 was extremely sensitive to cefotaxime, florfenicol, ciprofloxacin, cotrimoxazole, doxycycline, and gentamicin (inhibitory circle diameter > 20.00 mm) (Table 2). Images of antibiotic susceptibility analysis of *B. velezensis* K12 are shown in the Supplementary Materials (Figure S2).

### 3.3. In Vitro Bacteriostatic Activity of B. Velezensis K12

The fermentation broth of *B. velezensis* K12 exhibited varying inhibitory activity against seven indicator bacteria (*p* < 0.05). As the broth was diluted, its inhibitory potential decreased. Notably, the fermentation broth was highly sensitive to *E. coli* CVCC25922, *E. coli* K88, *Salmonella* CVCC519, *Staphylococcus aureus* CVCC1882, and *Vibrio parahaemolyticus* CICC23924, with a minimum inhibitory dilution of 1:10,000. The minimum inhibitory dilution for *Bacillus cereus* CICC21290 was 1:4000, and the minimum inhibitory dilution for *Clostridium perfringens* CVCC66 was 1:1000 (Table 3). The diameter of the *B. velezensis* K12 inhibition circle decreased linearly with the increasing dilution gradient of the indicator bacteria, except for *Staphylococcus aureus* CVCC1822 and *Bacillus cereus* CICC21290 (line *p*-value < 0.001). Images of the circle-of-inhibition diameter of the *B. velezensis* K12 bacterial solution against seven indicator bacteria are shown in the Supplementary Materials (Figure S3).

After isolating the organism and supernatant of *B. velezensis* K12, it was found that the organism exhibited bacteriostatic activity against all indicator bacteria (*p* < 0.05). In addition, the sterile supernatant demonstrated bacteriostatic activity against *E. coli* K88, *Bacillus cereus* CICC21290, *Clostridium perfringens* CVCC66, and *Vibrio parahaemolyticus* CICC23924 (*p* < 0.05). Notably, the inhibitory effect of whole bacteria was more potent than that of sediment, and the inhibitory effect of sediment was more substantial than that of the sterile supernatant (*p* < 0.05) (Table 4). Images of the circle-of-inhibition diameters of different components of *B. velezensis* K12 against seven indicator bacteria are shown in the Supplementary Materials (Figure S4). Furthermore, after inactivating *B. velezensis* K12, no inhibitory effect on the indicator bacterium *E. coli* K88 was observed (*p* < 0.05) (Table 5).

### 3.4. Whole-Genome Sequencing Results of B. velezensis K12

#### 3.4.1. Genome Composition of *B. velezensis* K12

**Figure 5:**
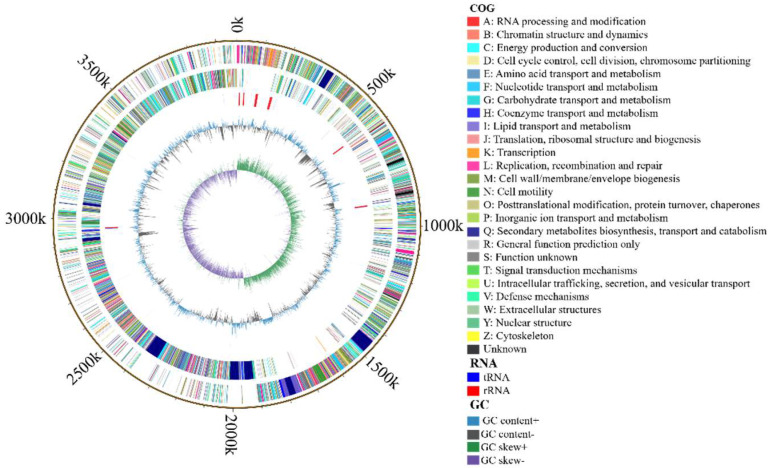
The genomic map of *B. velezensis* K12.

The genome of *B. velezensis* K12 comprises a circular sequence measuring 3,973,105 bp long, with an average GC content of 46.69% (Table 6). It includes 3913 protein-coding genes, which constitute 88.68% of the genome, and 97 ncRNAs, 86 tRNAs, and 27 rRNAs. The genome of *B. velezensis* K12 included 121 long terminal repeats, 35 long interspersed nuclear elements, 17 short interspersed elements, 19 prophages, and 21 genomics islands. The distribution of genes predicted by *B. velezensis* K12 gene islands is mapped in the Supplementary Materials (Figure S5). No CRISPR elements were detected in the genome. The circular genome diagram illustrates various features. Starting from the innermost ring and moving outward, the following features are noticed: GC skew in the first ring; GC content in the second ring; tRNA and rRNA positions in the third and fourth rings, respectively [61]; CDSs on the positive and negative strands of the fifth and sixth rings, with colors indicating different COG classifications; and the chromosomal karyotype in the outermost ring (Figure 5). The complete genome sequence of strain *B. velezensis* K12 has been submitted to GenBank (NCBI) with accession number CP171691.

#### 3.4.2. Functional Annotation of *B. velezensis* K12 Genome

**Figure 6:**
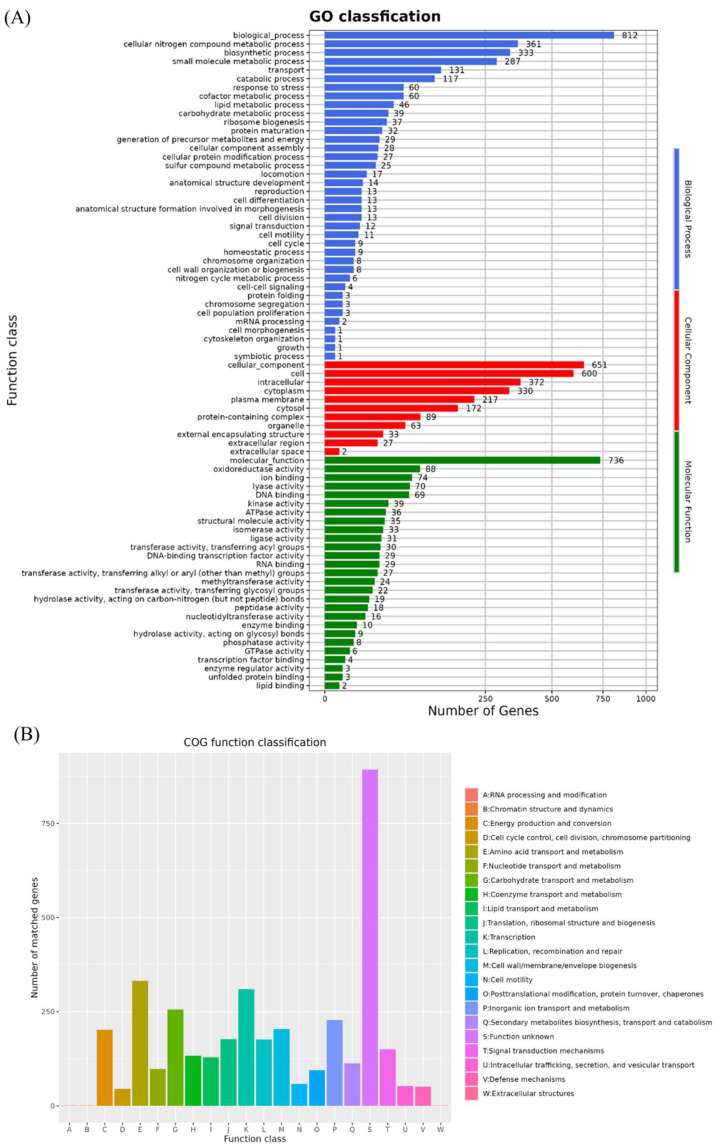
Functional annotation of GO and COG database. (**A**) GO annotation of *B. velezensis* K12. (**B**) COG annotation of *B. velezensis* K12.

By comparing the amino acid sequences of the *B. velezensis* K12 genome to the GO database, we identified 2199 functional genes. Among these, 736 genes were related to molecular functions, 812 to biological processes, and 651 to cellular components (Figure 6A). Specifically, in terms of molecular functions, 88 genes were classified as having oxidoreductase activity and 74 as having ion-binding functions. Within biological processes, 361 genes were classified as being involved in cellular nitrogen compound metabolism and 331 in biosynthesis. For cellular components, 600 genes were classified as cellular and 372 as intracellular.

A total of 3706 protein-coding genes in the *B. velezensis* K12 genome were annotated in the COG database (Figure 6B). Among these, the largest group consisted of 893 genes with unknown functions. The functional categories with high numbers of annotated genes were as follows: amino acid transport and metabolism (E) with 332 genes, transcription (K) with 310 genes, and carbohydrate transport and metabolism (G) with 256 genes.

**Figure 7:**
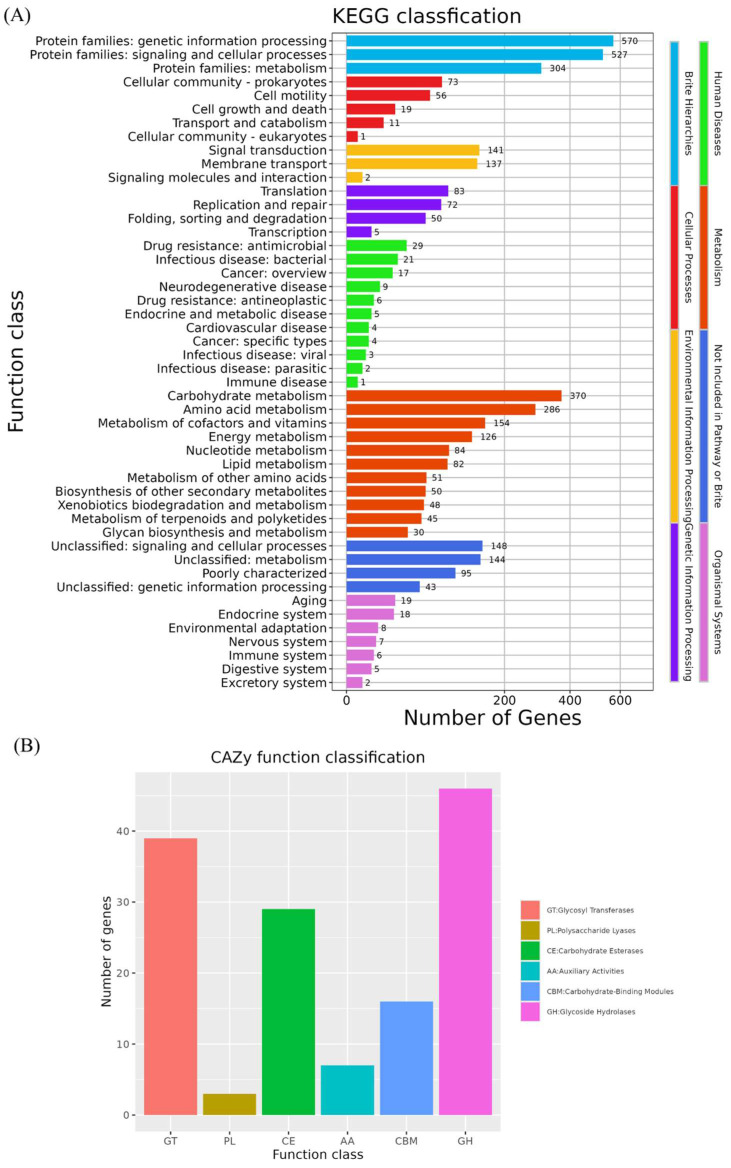
Functional annotation of KEGG and CAZy database. (**A**) KEGG annotation of *B. velezensis* K12. (**B**) CAZy annotation of *B. velezensis* K12.

The genome of *B. velezensis* K12 contains 3973 genes annotated through the KEGG database (Figure 7A). Among them, 570 genes were related to genetic information processing, making this the most represented category. This category was followed by annotations related to environmental information processing, genetic information processing, cellular processes, human diseases, and organic systems. Other frequently annotated functions included signaling and cellular processes (527 genes), carbohydrate metabolism (370 genes), protein family metabolism (307 genes), and amino acid metabolism (286 genes).

The genome of *B. velezensis* K12 has 140 genes annotated in the CAZy database (Figure 7B). The data indicated the presence of 46 genes associated with glycoside hydrolases (GHs) and 39 genes associated with glycosyltransferases (GTs). In addition, 29 genes were associated with carbohydrate esterases (CEs), and 16 were linked to carbohydrate-binding modules (CBMs). There are seven genes associated with auxiliary activities (AAs) and three genes associated with polysaccharide lyases (PLs).

CARD is the most widely used and comprehensive database of bacterial drug-resistant genes. Through the annotation of the CARD database, with IDENTITY ≥ 80% as the screening condition, the genome of *B. velezensis* K12 had six drug-resistant genes, among which *lmrB*, *clbA*, *ykkD*, and *rpoB* genes had multi-drug resistance, and it was hypothesized that *B. velezensis* K12 might have specific drug resistance (Table 7).

The genome of *B. velezensis* K12 had four virulence factors, including the *hlyIII* virulence gene related to exotoxin, the *dhbE* virulence gene related to nutrient metabolism, and *dep/capD* and *capB* related to immune regulation, as annotated by the VFDB database with IDENTITY ≥ 80% as the screening condition (Table 8).

Thirteen secondary metabolite gene clusters were identified in the genome of strain *B. velezensis* K12 by AntiSMASH 7.0 software, of which 10 were associated with synthesizing known repressor secondary metabolites (Table 9). Of these, four gene clusters were predicted to have 95%, 100%, 100%, and 100% synthetic similarity to fengycin, surfactin, bacillothiazol, and bacillibactin in non-ribosomal peptides (NRPs), respectively. Three clusters were predicted to have 100% synthetic similarity to the polyketide synthases (PKSs) in the synthesis of macrolactin H, bacillaene, and difficidin; two clusters of genes had 100% and 91% similarity to lanthipeptide and plantazolicin in ribosomal synthesis and post-translationally modified peptides (RiPPs), respectively; and one cluster of genes had 100% similarity to bacillinolysin. In addition, there are three unknown gene clusters in the *B. velezensis* K12 genome, including two terpenes and one polyketide (T3PKS), suggesting that the *B. velezensis* K12 gene cluster may synthesize new bacteriostatic substances.

Whole-genome sequence analysis showed that the *B. velezensis* K12 genome contains several putative probiotic genes for acid tolerance, bile salt tolerance, adhesion aggregation, and antioxidant resistance that may help *B. velezensis* K12 to survive in the gastrointestinal environment (Table 10).

## 4. Discussion

The dire resistance to traditional feed antibiotics has accelerated the pursuit of antibiotic alternatives. Recently, probiotic products have garnered increasing attention in the livestock and poultry industries. In 2019, the European Food Safety Authority (EFSA) granted *B. velezensis* Qualified Presumption of Safety (QPS) status, proving that it does not have toxicological potential and has aminoglycoside production capacity [62]. Nonetheless, *B. velezensis* has not received substantial recognition or endorsement as a safe microbial feed additive, likely due to the paucity of data regarding its biosafety and probiotic properties at phenotypic and genetic levels [63]. This study assessed the probiotic potential and safety profile of *B. velezensis* K12 through in vitro experiments complemented by genomic analyses.

Antimicrobial properties against pathogenic bacteria are the main selection criteria for probiotics in animal feed and nutrition [64]. In this study, the strain K12, with a strong inhibitory effect against bacteria, was screened and identified as *Bacillus velezensis*. *Escherichia coli*, *Salmonella* spp., *Staphylococcus aureus,* and *Clostridium perfringens* are common pathogens in poultry [65]. *Vibrio parahaemolyticus* is the most critical aquatic pathogen in the genus *Vibrio* and poses a significant hazard to aquaculture [66]. *Bacillus cereus* is one of the essential human enteric pathogens [67]. *B. velezensis* K12 showed varying degrees of inhibitory effects on the seven pathogenic bacteria mentioned above, and its whole bacteria exhibited greater inhibitory potential than sediments and supernatants, with the inhibitory effect ceasing after inactivation. These findings hint that the bacteriostatic effect of *B. velezensis* K12 may come from the secretion of heat-intolerant bacteriostatic substances by live bacteria. These inhibitory substances may be NRPs, PKS, bacilysin, and RiPPs (Table 9). Among them, fengycin, difficidin, bacilysin, macrolactin, and bacillibactin have been reported to have broad-spectrum antibacterial effects [68,69,70,71]. These antibacterial compounds have been reported to operate in three mechanisms: cell wall disruption, the control of critical cell machinery DNA and RNA, and the inhibition of enzyme activity [11].

Probiotics must endure the oxidative challenges present in the gastrointestinal tract to extend benefits to the host [72]. The whole-genome sequencing revealed genes associated with acid and bile tolerance in the *B. velezensis* K12 genome (Table 10). Based on the annotation results, *B. velezensis* K12 utilizes eight ATP synthase *atp* genes, organized in an operon to sustain H^+^ homeostasis in acidic conditions by hydrolyzing ATP to extrude protons from the cytoplasm [63]. The Na^+^/H^+^ antiporter and Na^+^ (K^+^, Li^+^, and/or alkali)/H^+^ antiporter genes play an essential role in Na^+^ resistance, pH homeostasis, and osmoregulation, enabling bacterial survival in acidic environments [73]. The enzyme bile salt hydrolase was encoded by *bsh* genes (*bshA*, *bshB1*, *bshC*), which play an essential role in the cholesterol-lowering effect by hydrolyzing conjugated bile salts into amino acids and deconjugated bile salts [74]. In addition, the genes encoding *dnaK*, *dnaJ*, *pryG*, *eno,* and *clp* protease chaperones are up-regulated in acid shock, facilitating the strain’s tolerance to thermal and osmotic stress while aiding in the repair of damaged proteins [75,76]. By measuring the growth of the strain under different conditions, it was found that *B. velezensis* K12 was not adapted to the artificial gastric fluid environment compared to the artificial intestinal fluid, which is consistent with the results of *B. velezensis* K12 intolerance in environments with pH below 3.5. In the future, *B. velezensis* K12 could be delivered to the intestine with the help of embedding technology or enteric formulations.

Mucus adherence and penetration potential are crucial probiotic characteristics facilitating the transitory colonization of the host intestinal epithelial surface and the competitive exclusion of pathogens [77]. The *B. velezensis* K12 genome encodes numerous reported surface proteins implicated in adhesion and aggregation (Table 10), including mucus adhesion domain protein (*lspA*, *glnH*, *tuf*), fibronectin-binding protein (*fbp*), *Bacillus subtilis* matrix protein TasA (*tasA* 1), teichoic-acid (*tagO*), and exopoly saccharides (*eps*, *lip*, *galE*) [77,78,79,80,81]. Multiple investigations have demonstrated that *Bacillus velezensis* has probiotic properties that enhance the antioxidant capacity of the host [82,83]. In this study, we identified numerous genes within *B. velezensis* K12 that encode glutathione peroxidase (*bshA*), alkyl hydroperoxide reductase (*ahp*), catalases (*kat*), and superoxide dismutase (*sodF*), which also confirmed the antioxidant properties of *Bacillus velezensis*.

Probiotic candidates must be analyzed for safety, including assessments of hemolysis, resistance, and virulence. The surfactin and *hlyIII* genes related to hemolysis were annotated in the *B. velezensis* K12 genome. As an antimicrobial peptide, surfactin exerts hemolytic activity by modifying the integrity of cell membranes [84]. It produces surface-active effects that are not pathogenic. The *hlyIII* gene encoding hemolysin alone did not determine bacterial pathogenicity and was not found on transferable plasmids. It has been shown that *B. velezensis* FCW2 MCC4686, which carries the *hlyIII* gene, did not cause mortality or disease symptoms in the zebrafish model, suggesting that the strain is non-pathogenic [59]. In addition, in vitro hemolysis tests showed that *B. velezensis* K12 did not induce the hemolysis of hemocytes on sheep blood agar and was not hemolytic. Antibiotic resistance genes associated with antibiotics, such as aminoglycoside (*rpsE*) and tetracycline (*rpoB*) in the *B. velezensis* K12 genome, were identified by CARD analysis. However, in vitro antibiotic susceptibility testing showed that *B. velezensis* K12 was susceptible to six antibiotics, including gentamicin and tetracycline. This suggests that the *B. velezensis* K12 resistance genes may lead to an inherent resistance mechanism during microbial formation, but this is not generally transferred between organisms [85]. Although the genome of *B. velezensis* K12 has four virulence factors annotated by the VFDB, they are not considered genuinely harmful. *B. velezensis* TS5, which carries virulence genes similar to *B. velezensis* K12, related to immunomodulatory and nutritional/metabolic factors, is safe in mice [86]. In addition, common virulence genes of *Bacillus* enterotoxins, including hemolysin A (*hlyA*), hemolysin II (*hlyII*), hemolysin BL (*hbl*), non-hemolytic enterotoxin (*Nhe*), enterotoxin T (*bceT*), and cytotoxin K (*CytK*), were not found in the *B. velezensis* K12 genome [87,88]. The above safety analysis shows that *B. velezensis* K12 has a favorable in vitro safety profile. However, to fully understand its safety and effects in vivo, additional animal studies are necessary in the future.

## 5. Conclusions

These in vitro and genomic studies provide valuable insights into the essential properties of *B. velezensis* K12. This strain possesses potent bacteriostatic properties and has exhibited safety in vitro. However, the specific mechanism of bacterial inhibition needs to be further investigated. *B. velezensis* K12 was sensitive to six antibiotics and had acid tolerance. Genome mining revealed that *B. velezensis* K12 contains genes encoding host intestinal adhesion and antioxidants. The results of this study prove that *B. velezensis* K12 is a promising candidate for further probiotic development.

## Figures and Tables

**Table 1:** Tolerance analysis of *B. velezensis* K12 to simulated gastrointestinal fluids.

	Simulated Gastrointestinal Fluids	Time/h		Survival Rate/%
		0	3	
Bacterial concentration (CFU/mL)	Simulated gastric fluid	1.1 × 10^8^	1.1 × 10^4^	0.01%
	Simulated intestinal fluid	8.6 × 10^7^	3.5 × 10^7^	40.70%

**Table 2:** Antibiotic susceptibility analysis of *B. velezensis* K12.

Antibiotics	Inhibitory Circle Diameter/mm
Florfenicol	29.08 ± 1.17
Ciprofloxacin	27.69 ± 0.11
Cotrimoxazole	22.94 ± 0.04
Gentamycin	23.54 ± 0.11
Doxycycline	21.59 ± 1.04
Cefotaxime	22.33 ± 5.43

**Table 3:** Circle-of-inhibition diameter of *B. velezensis* K12 bacterial solution against seven indicator bacteria (mm).

Dilution Factor	*E. coli* CVCC25922	*E. coli* K88	*Staphylococcus aureus* CVCC1822	*Salmonella* CVCC519	*Bacillus* *cereus* CICC21290	*Clostridium perfringens* CVCC66	*Vibrio parahaemolyticus* CICC23924
Control	8.00 ± 0.00 ^e^	8.00 ± 0.00 ^d^	8.00 ± 0.00 ^g^	8.00 ± 0.00 ^f^	8.00 ± 0.00 ^e^	8.00 ± 0.00 ^d^	8.00 ± 0.00 ^g^
10	22.06 ± 2.24 ^a^	18.11 ± 2.84 ^a^	20.89 ± 0.19 ^a^	19.50 ± 0.44 ^a^	17.00 ± 0.00 ^a^	13.11 ± 0.19 ^a^	21.02 ± 0.03 ^a^
100	18.89 ± 1.92 ^b^	16.66 ± 2.32 ^ab^	18.36 ± 0.70 ^b^	17.06 ± 0.42 ^b^	14.11 ± 0.19 ^b^	11.56 ± 0.51 ^b^	19.50 ± 0.50 ^b^
1000	16.33 ± 2.08 ^c^	15.23 ± 2.17 ^abc^	14.33 ± 0.44 ^c^	15.45 ± 0.39 ^c^	12.67 ± 0.34 ^c^	10.89 ± 0.35 ^c^	17.83 ± 0.58 ^c^
4000	14.17 ± 0.73 ^cd^	14.19 ± 2.01 ^abc^	13.07 ± 0.09 ^d^	13.50 ± 0.44 ^d^	10.94 ± 0.10 ^d^	8.00 ± 0.00 ^d^	16.83 ± 0.29 ^d^
8000	13.28 ± 1.11 ^d^	12.72 ± 2.33 ^bc^	12.06 ± 0.05 ^e^	13.06 ± 0.10 ^d^	8.00 ± 0.00 ^e^	8.00 ± 0.00 ^d^	15.67 ± 0.58 ^e^
10,000	12.44 ± 0.96 ^d^	11.33 ± 3.51 ^cd^	11.00 ± 0.00 ^f^	11.78 ± 0.39 ^e^	8.00 ± 0.00 ^e^	8.00 ± 0.00 ^d^	14.50 ± 0.87 ^f^
Line *p*-value	<0.001	<0.001	0.057	<0.001	0.256	<0.001	<0.001

^a–g^ Superscripts without common letters in the same column denote significant differences (*p* < 0.05).

**Table 4:** Circle-of-inhibition diameters of different components of *B. velezensis* K12 against seven indicator bacteria (mm).

Sample	*E. coli* CVCC25922	*E. coli* K88	*Staphylococcus aureus* CVCC1822	*Salmonella* CVCC519	*Bacillus* *cereus* CICC21290	*Clostridium perfringens* CVCC66	*Vibrio parahaemolyticus* CICC23924
Sterile water	8.00 ± 0.00 ^b^	8.00 ± 0.00 ^c^	8.00 ± 0.00 ^b^	8.00 ± 0.00 ^c^	8.00 ± 0.00 ^d^	8.00 ± 0.00 ^c^	8.00 ± 0.00 ^d^
Whole bacteria	16.83 ± 1.61 ^a^	16.20 ± 0.26 ^a^	16.83 ± 1.61 ^a^	18.67 ± 1.15 ^a^	18.78 ± 0.84 ^a^	13.00 ± 1.00 ^a^	17.83 ± 0.29 ^a^
Supernatant	8.00 ± 0.00 ^b^	15.03 ± 0.50 ^b^	8.00 ± 0.00 ^b^	8.00 ± 0.00 ^c^	12.11 ± 0.19 ^c^	11.00 ± 1.00 ^b^	14.87 ± 0.32 ^c^
Sediment	15.50 ± 1.50 ^a^	15.07 ± 1.01 ^b^	15.50 ± 1.50 ^a^	17.17 ± 1.04 ^b^	15.56 ± 0.51 ^b^	11.33 ± 1.15 ^b^	17.37 ± 0.23 ^b^
LB	8.00 ± 0.00 ^b^	8.00 ± 0.00 ^c^	8.00 ± 0.00 ^b^	8.00 ± 0.00 ^c^	8.00 ± 0.00 ^d^	8.00 ± 0.00 ^c^	8.00 ± 0.00 ^d^
PBS	8.00 ± 0.00 ^b^	8.00 ± 0.00 ^c^	8.00 ± 0.00 ^b^	8.00 ± 0.00 ^c^	8.00 ± 0.00 ^d^	8.00 ± 0.00 ^c^	8.00 ± 0.00 ^d^

^a–d^ Superscripts without common letters in the same column denote significant differences (*p* < 0.05).

**Table 5:** Circle-of-inhibition diameters of *B. velezensis* K12 inactivated supernatant (mm).

Samples	*E. coli* K88
Whole bacteria	16.56 ± 0.51 ^a^
Boiled	8.00 ± 0.00 ^b^
High-pressure treatment	8.00 ± 0.00 ^b^
LB	8.00 ± 0.00 ^b^

^a,b^ Superscripts without common letters in the same column denote significant differences (*p* < 0.05).

**Table 6:** Genome composition of *B. velezensis* K12.

Genome	Value
Seq length (bp)	3,973,405
Seq type	Circular
G + C content (%)	46.69
N20 length (bp)	28,206
N50 length (bp)	9802
N90 length (bp)	1825
Max length (bp)	174,061
Min length (bp)	1
Total number of genes	4123
Number of coding sequences (CDS)	3913
Average length (bp)	900.47
Gene/genome (%)	88.68
Prophages	19
Genomic islands	21
Number of RNA genes	210

**Table 7:** Results of CARD annotation of *B. velezensis* K12.

Gene Number	Resistant Gene	Antibiotic Resistance	Resistance Mechanism
chr_263	*lmrB*	lincosamide antibiotic; nucleoside antibiotic	Antibiotic efflux
chr_519	*clbA*	lincosamide antibiotic; oxazolidinone antibiotic; phenicol antibiotic; pleuromutilin antibiotic; streptogramin A antibiotic; streptogramin antibiotic	Antibiotic target alteration
chr_1328	*ykkD*	aminoglycoside antibiotic; phenicol antibiotic; tetracycline antibiotic	Antibiotic efflux
chr_124	*EF-Tu*	elfamycin antibiotic	Antibiotic target alteration
chr_118	*rpoB*	peptide antibiotic; rifamycin antibiotic	Antibiotic target alteration; antibiotic target replacement
chr_142	*rpsE*	aminoglycoside antibiotic	Antibiotic target alteration

**Table 8:** Results of VFDB annotation of *B. velezensis* K12.

Gene Number	VF Id (gb Number)	Virulence Gene	VF Function	Identity/%
chr 1907	VFG016303 (gb|NP 389723)	*dep/capD*	Immune modulation	84.615
chr 2046	VFG016235 (gb|WP 003182805)	*hlyIII*	Exotoxin	80.189
chr 2967	VFG050021 (gb|NP 391078)	*dhbE*	Nutritional/Metabolic factor	81.051
chr 3389	VFG016299 (gb|NP 391471)	*capB*	Immune modulation	93.13

**Table 9:** The predicted gene clusters for secondary metabolites of *B. velezensis* K12.

Type	Start	End	Most Similar Known Cluster	Similarity
lanthipeptide-class-iii	193,784	216,399	andalusicin A; andalusicin B RiPP:Lanthipeptide	100%
NRPS	314,996	380,403	surfactin NRP:Lipopeptide	95%
RRE-containing, LAP	708,772	731,949	plantazolicin RiPP:LAP	91%
terpene	1,097,917	1,118,657	-	-
transAT-PKS	1,421,088	1,509,330	macrolactin H Polyketide	100%
transAT-PKS, T3PKS, NRPS	1,731,593	1,841,698	bacillaene Polyketide + NRP	100%
NRPS, transAT-PKS, betalactone	1,898,733	2,036,597	fengycin NRP	100%
terpene	2,061,882	2,083,765	-	-
T3PKS	2,143,224	2,184,324	-	-
transAT-PKS	2,300,233	2,406,424	difficidin Polyketide	100%
NRPS	2,892,056	2,942,291	bacillothiazol	100%
NRP-metallophore, NRPS, RiPP-like	3,039,145	3,090,934	bacillibactin NRP	100%
Other	3,624,730	3,666,148	bacilysin other	100%

**Table 10:** Putative probiotic genes in the genome of *B. velezensis* K12.

Functional Category	Gene	Description/Function	Gene Number
Acid stress	*atp*	ATP synthase	chr 3493, chr 3495, chr 3487, chr 3488, chr 3489, chr 3490, chr 3492, chr 3494
	*atpH*	F(1)F(0)-ATP synthase	chr 3491
	*nhaC*	Na^+^/H^+^ antiporter	chr 2382
	*nhaC 1*	Na^+^/H^+^ antiporter	chr 530
	*mleN*	Na^+^/H^+^ antiporter	chr 2225
	*yuiF*	Na^+^/H^+^ antiporter	chr 2973
	*ycgA*	Na^+^/H^+^ antiporter	chr 291
	*-*	Na^+^/H^+^ antiporter	chr 904
	*mrp*	Na^+^ (K^+^, Li^+^ and/or alkali)/H^+^ antiporter	chr 164, chr 2931, chr 2932, chr 2933, chr 2934, chr 2935, chr 2936, chr 2937
Bile tolerance	*bshC*	Glucosaminyl-malate cysteine ligase	chr 1545
	*bshA*	*N*-acetyl-alpha-D-glucosaminyl L-malate synthase	chr 2114
	*bshB1*	Acetylglucosamine-malate deacetylase	chr 2115
	*pyrG*	CTP synthase	chr 3523
	*dnaK*	Chaperone protein	chr 2440
	*dnaJ*	Chaperone protein	chr 2439
	*eno*	Enolase C/Enolase N	chr 3202
	*clp*	CLP protease	chr 97, chr 1386, chr 2596, chr 3264
	*oppA*	ABC transporter substrate-binding protein	chr 1159
	*nagB*	Glucosamine-6-phosphate deaminase	chr 3301
	*-*	Sodium bile acid symporter	chr 526
Adhesion	*fbp*	Firmicute fructose-1,6-bisphosphatase	chr 3790, chr 3797
	*eps*	Glycosyltransferases	chr 3232, chr 3233, chr 3234, chr 3235, chr 3236, chr 3237, chr 3238, chr 3239, chr 3240, chr 3241, chr 3242, chr 3243
	*lip*	Lipase	chr 266
	*galE*	NAD(P)-dependent epimerase dehydratase	chr 1228
	*lspA*	Peptidase	chr 1575
	*glnH*	Bacterial solute-binding protein	chr 2519
	*tuf*	GTP EFTU	chr 124
	*tasA 1*	Cell division protein	chr 2356
	*tagO*	UDP-*N*-acetylglucosamine-1-phosphate transferase	chr 3351
Oxidative	*kat*	Catalase	chr 914, chr 3685, chr 3712
	*ahp*	Alkyl hydroperoxide reductase	chr 3786, chr 3787
	*bsaA*	glutathione peroxidase	chr 2056
	*sodF*	Superoxide dismutase	chr 1962

## Data Availability

All data are contained within the article.

## Supplementary Materials

The following supporting information can be downloaded at: https://www.mdpi.com/article/10.3390/ani15060798/s1, Figure S1: Hemolysis analysis of *B. velezensis* K12. Figure S2: Antibiotic susceptibility analysis of *B. velezensis* K12. Figure S3: Circle-of-inhibition diameter of *B. velezensis* K12 bacterial solution against seven indicator bacteria. Figure S4: Circle-of-inhibition diameters of different components of *B. velezensis* K12 against seven indicator bacteria. Figure S5: Map of gene distribution on genetic islands of *B. velezensis* K12.

## Abbreviations

The following abbreviations are used in this manuscript:

ISAPP	International Scientific Association for Probiotics and Prebiotics
*E. coli*	*Escherichia coli*
LB	Luria–Bertani
CLSI	American Society for Clinical Laboratory Standardization
ANI	Average Nucleotide Identity
UBCG	Up-to-Date Bacterial Core Gene
KEGG	Kyoto Encyclopedia of Genes and Genomes
COGs	Clusters of Orthologous Groups
GO	Gene Ontology
CAZy	Carbohydrate-Active Enzymes database
CARD	Comprehensive Antibiotic Research Database
VFDB	Virulence Factors of Pathogenic Bacteria Database
antiSMASH	Antibiotics and Secondary Metabolite Analysis Shell
ANOVA	One-way analysis of variance
NCBI	National Center for Biotechnology Information
NRPs	Non-ribosomal peptides
PKS	Polyketide synthases
RiPPs	Ribosomal synthesis and post-translationally modified peptides
EFSA	European Food Safety Authority
QPS	Qualified Presumption of Safety
