# Supplementary material for: Comprehensive Phenotypic Characterization and Genomic Analysis Unveil the Probiotic Potential of Bacillus velezensis K12
Source: Animals (Basel). 2025 Mar 11;15(6):798. doi: 10.3390/ani15060798 (PMC11939506; doi:10.3390/ani15060798)
Supplement: Supplementary file 1 [file animals-15-00798-s001.zip › animals-3472127-supplementary.pdf]

Comprehensive Phenotypic Characterization and Genomic Analysis Unveil the Probiotic Potential of *Bacillus velezensis* K12

Yingying Tang [1], Tian Li [2], Yihong Huang [1], Liangliang Wu [3], Xiaobo Liu [3], Ruichao Yue [4,*] and Jianmin Yuan [1,*]

[1] State Key Laboratory of Animal Nutrition and Feeding, College of Animal Science and Technology, China Agricultural University, Beijing 100193, China; sy20243041032@cau.edu.cn (Y.T.); huangyh@cau.edu.cn (Y.H.);
[2] College of Animal Science and Technology & College of Veterinary Medicine, Zhejiang A&F University, Hangzhou, 310000, China; hsjimmyf@hotmail.com (T.L.)
[3] Ningxia Eppen Biotech Co., Ltd., Yinchuan 750100, China; wuliangliang@eppen.com.cn (L.W.); liuxiaobo@eppen.com.cn (X.L.)
[4] Department of Basic Veterinary Medicine, College of Veterinary Medicine, China Agricultural University, Beijing, 100193, China; r_yue@cau.edu.cn (R.Y.)
* Correspondence: r_yue@cau.edu.cn (R.Y.); yuanjm@cau.edu.cn (J.Y.);

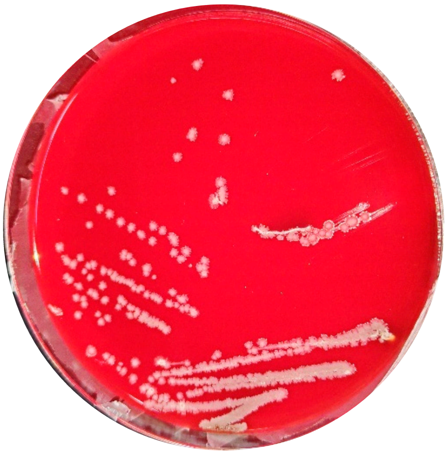

Figure S1. Hemolysis analysis of *B. velezensis* K12.

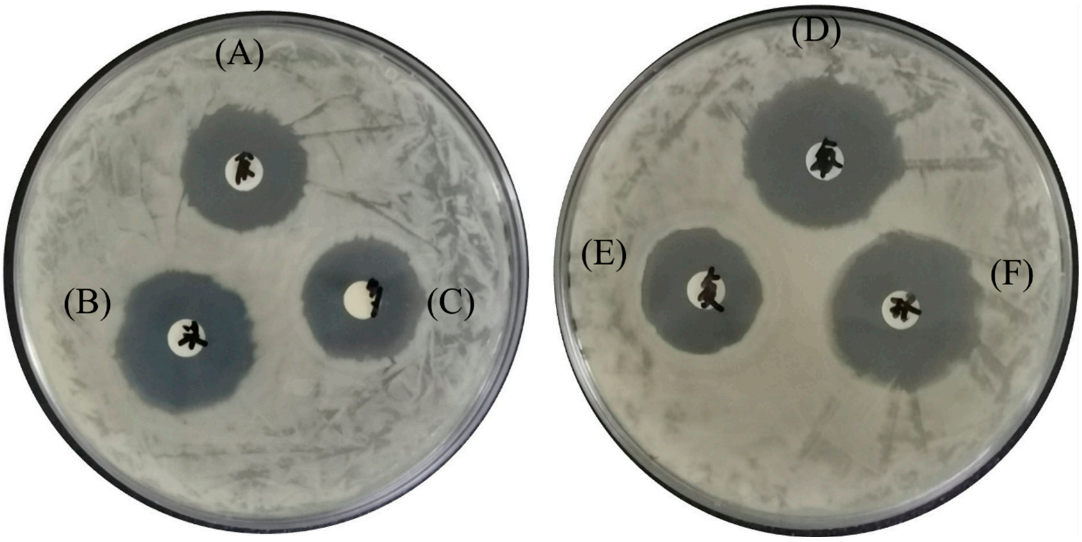

Figure S2. Antibiotic susceptibility analysis of *B. velezensis* K12. (A) Gentamycin. (B) Cefotaxime. (C) Doxycycline. (D) Florfenicol. (E) Cotrimoxazole. (F) Ciprofloxacin.

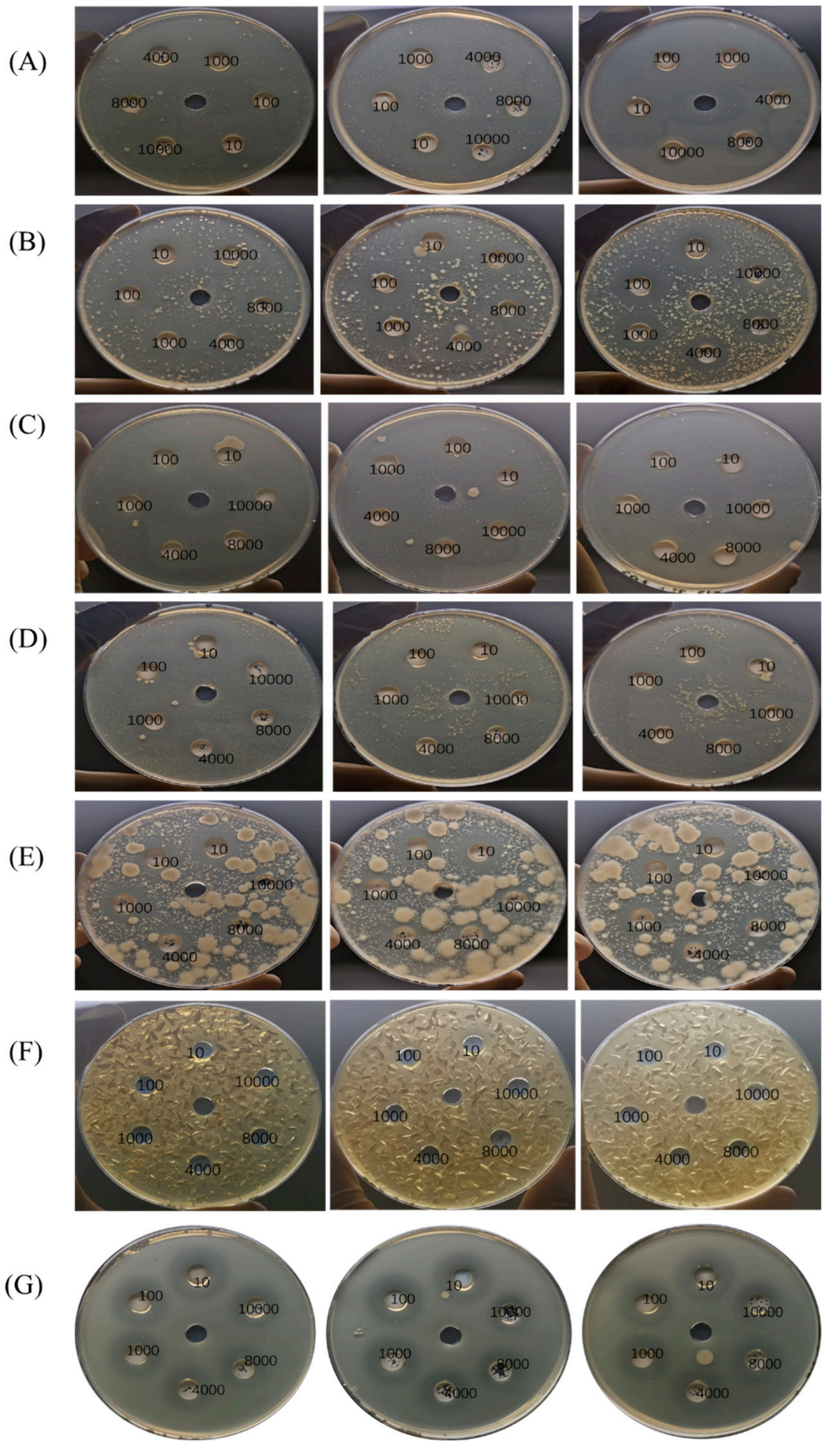

Figure S3. Circle-of-inhibition diameters of *B. velezensis* K12 bacterial solution against seven indicator bacteria. (A) *E. Coli* CVCC25922. (B) *E. coli* K88. (C) *Staphylococcus aureus* CVCC1822. (D) *Salmonella* CVCC519. (E) *Bacillus cereus* CICC21290. (F) *Clostridium perfringens* CVCC66. (G) *Vibrio parahaemolyticus* CICC23924.

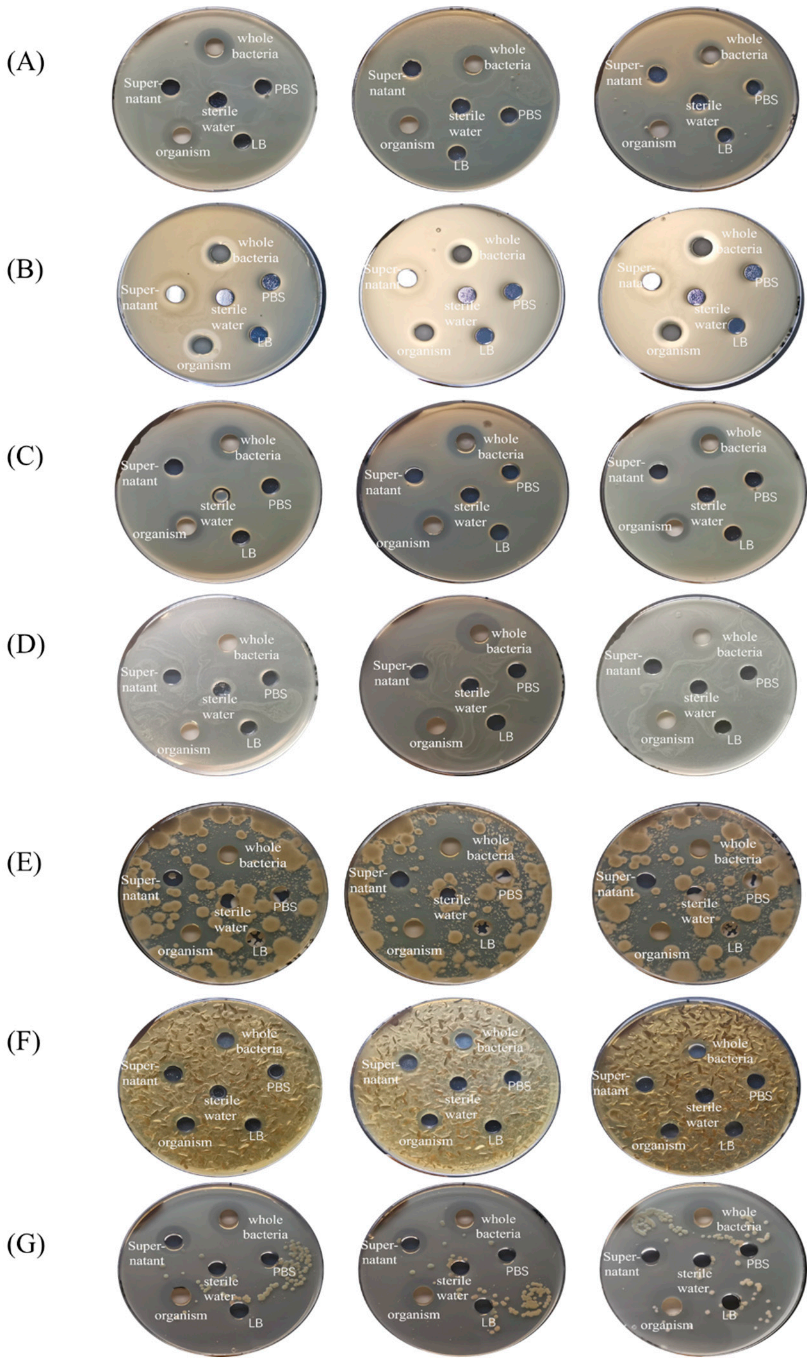

Figure S4. Circle-of-inhibition diameters of different components of B. velezensis K12 against seven indicator bacteria. (A) *E. Coli* CVCC25922. (B) *E. coli* K88. (C) *Staphylococcus aureus* CVCC1822. (D) *Salmonella* CVCC519. (E) *Bacillus cereus* CICC21290. (F) *Clostridium perfringens* CVCC66. (G) *Vibrio parahaemolyticus* CICC23924.

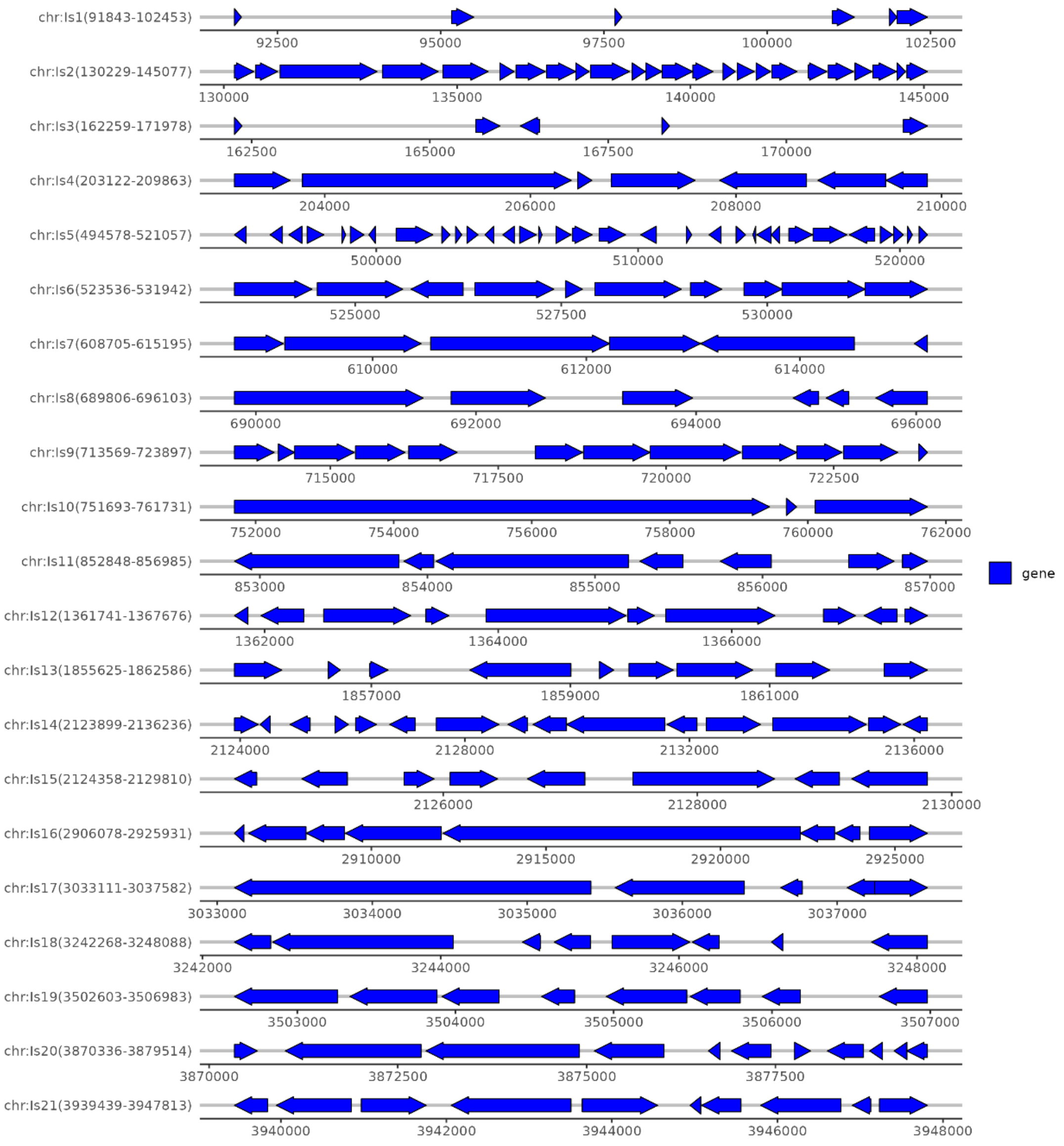

Figure S5. Map of gene distribution on genetic islands of *B. velezensis* K12.
